# Development and evaluation of pupal color‐based genetic sexing strains in *Anastrepha obliqua* (Diptera: Tephritidae)

**DOI:** 10.1111/1744-7917.70163

**Published:** 2025-09-08

**Authors:** Daisy P. Cárdenas‐Enríquez, Víctor García‐Martínez, Jorge Ibáñez‐Palacios, Brenda Torres‐Huerta, Maria F. Ruiz‐Pérez, José S. Meza

**Affiliations:** ^1^ Programa Operativo Moscas SADER/SENASICA‐IICA Metapa de Domínguez Chiapas Mexico

**Keywords:** chromosomal translocation, genetic sexing strain, mass rearing, recessive mutation, sterile insect technique

## Abstract

*Anastrepha obliqua*, a neotropical pest widely distributed in the Americas, attacks mango and other tropical fruits. In Mexico, it is controlled through integrated pest management, using the Sterile Insect Technique (SIT) as a main component. The applicability of SIT is significantly improved with the use of genetic sexing strains (GSS) that allow the possibility to release exclusively sterile males, the primary component of the technique. This study reports the isolation and characterization of two pupal mutations: *black pupae* (*bp*) and *sphere pupae* (*sp*), allowing for the first time the development of a genetic sexing system based on pupal color in this species. Inheritance analyses from reciprocal crosses between wild‐type and mutant individuals showed F_2_ phenotypic segregation consistent with a recessive Mendelian inheritance pattern, and linkage analysis indicated that the *bp* and *sp* loci are in separate chromosomes. Using the *bp* mutation, two GSS were developed through gamma irradiation [T(Y;*bp* ^+^)/*bp*‐22](GSS‐22) and ethyl methanesulfonate treatment [T(Y;*bp^+^*)/*bp*‐354](GSS‐354). Both GSS exhibited sex‐specific pupal differentiation but displayed a high frequency of recombinants. Despite an initial reduction in biological fitness, GSS‐22 demonstrated greater genetic stability and a lower frequency of recombinants than GSS‐354. Discrepancies between cytogenetic and genomic data, particularly regarding the localization of the gene responsible for the black pupae phenotype, underscore the need to integrate polytene chromosome and genomic analyses to characterize these translocations and improve GSS stability precisely. These results represent a breakthrough in the creation of genetic tools for the management of *A. obliqua* control.

## Graphical abstract

Two spontaneous mutations in pupae of the West Indian fruit fly *Anastrepha obliqua*, named *bp* and *sp*, were isolated and studied. The *bp* mutation was selected, and using two types of Y‐autosoma l translocation inducers and an appropriate crossing scheme, two genetically sexed strains based on pupa l color were developed for the first time in this species, in which males emerge from wild‐type brown pupae and females from black pupae. One of these strains was obtained by exposure to gamma radiation and the other to methyl ethane sulfonate.

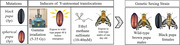

## Introduction

Fruit flies of the genus *Anastrepha* Schiner (Diptera: Tephritidae) are endemic to the Americas, and include more than 250 species, distributed from North America (southern Florida, Rio Grande Valley in Texas, Mexico) to southern Argentina (Aluja, 1994; Hernández‐Ortiz *et al.*, 2012; Norrbom & Korytkowski, 2012). These include *Anastrepha fraterculus* (Wiedemann 1830), *Anastrepha grandis* (Macquart 1846), *Anastrepha ludens* (Loew 1873), *Anastrepha obliqua* (Macquart 1835), *Anastrepha serpentina* (Wiedemann 1830), *Anastrepha striata* (Schiner 1868), and *Anastrepha suspensa* (Loew 1862), which are considered species of economic importance, as they directly affect the mango and citrus industry and have a wide host range (Bacca *et al.*, 2017; Degracia *et al.*, 2023). *A. obliqua*, commonly known as the West Indian fruit fly, is one of the most polyphagous species of this genus and is considered to be the second most economically important species in Mexico (Aluja & Mangan, 2008; Peña *et al.*, 2009; Liedo, 2016). It can cause economic yield losses ranging from 25% to 70%, and the presence of adults fruit flies in commercial orchards can lead to restrictions on fruit movement in low‐prevalence or temporarily fruit‐fly‐free areas (Aluja & Birke, 1993; Toledo *et al.*, 2005).

Area‐wide integrated pest management was first implemented in Mexico in 1992 to control *A. ludens* and *A. obliqua*, using the sterile insect technique (SIT) as the main component (Orozco‐Dávila *et al.*, 2017). The SIT is a species‐specific and environmentally safe genetic control method that involves mass‐rearing and sterilization of insects with irradiation before systematically releasing them into targeted areas to suppress, eradicate, or prevent the establishment of pest populations (Bourtzis & Vreysen, 2021). Some SIT applications have successfully used bisexual releases. However, the sole release of sterile males has proven to be more efficient and cost‐effective, as it maximizes matings with wild females and prevents fruit damage caused by oviposition from sterile females (Hendrichs *et al.*, 1995; Rendón *et al.*, 2004). Additionally, this approach reduces operational costs associated with rearing and release (Robinson, 2002a). The development of genetic sexing strains (GSS) makes possible the exclusive release of males in the field, and their construction requires two genetic components: (1) Selection and isolation of recessive morphological or biochemical markers in early stages such as the embryo, larva or pupa. (2) Induction of chromosomal rearrangements such as Y‐autosomal translocation, which involves the autosome carrying the selectable marker. This approach results in pseudo‐sexual dimorphism, with all males being wild type while only females display selectable marker (Alphey, 2007; Franz *et al.*, 2021). Successful examples include the VIENNA 8 strain of the Mediterranean fruit fly (*Ceratitis capitata*), a quarantine pest in Mexico and a species of global economic significance (Giunti *et al.*, 2023; Aluja *et al.*, 2024). This strain utilizes the *white pupae* (*wp*) marker and a *temperature‐sensitive lethal* (*tsl*) mutation to eliminate females at the embryonic and pupal stages (Augustinos *et al.*, 2017). Other notable examples are two *Anastrepha* species: *A. ludens* and *A. fraterculus*, both of which use the same *black pupae* morphological marker (*bp*), which allowing for the separation of females with black puparia from males with brown puparia (Zepeda‐Cisneros *et al.*, 2014; Meza *et al.*, 2020).

Currently, the application of SIT to *A. obliqua* does not allow the release of males only, a limitation that hinders operational optimization, reducing efficiency and increasing costs. To address this challenge, in 2017, the Genetics Department of the National Fruit Fly Program in Mexico launched a project to identify genetic markers in *Anastrepha* and develop a GSS using a classical genetic approach (Franz *et al.*, 2021). This study identified and characterized two novel genetic markers in *A. obliqua* and developed two GSSs using the *black pupae* (*bp*) visual marker. We induced chromosomal translocations by gamma irradiation and ethyl methanesulfonate (EMS) treatment, linking the autosomal wild type allele to the Y chromosome. Additionally, we assessed the biological attributes, genetic stability, and cytogenetic profiles of the resulting GSS to determine their viability and functionality in SIT application.

## Materials and methods

### Insect strain and rearing conditions

This study used a wild‐type (WT) strain of *A. obliqua* from the mutant bank of the Programa Operativo Moscas, SADER/SENASICA‐IICA, Genetics Department in Metapa de Domiguez, Chiapas, Mexico. Flies having the *black pupae* (*bp*) phenotype were isolated from a mass‐reared population after screening thousands of pupae. The *sphere pupae* (*sp*) phenotype was isolated from a small WT colony. Wild type flies and the two mutant lines, *bp* and *sp*, were maintained under controlled conditions (70%–80% relative humidity, 26 °C, and a 12 : 12 light/dark photoperiod). Larvae were reared on an artificial diet consisting of corncob fractions (16.3%,) sugar (9.3%), yeast (7.1%), corn flour (5.4%), citric acid (0.45 %), sodium benzoate (0.41%), Nipagin (0.2%), guar gum (0.05%), and water (60.8) (Pascacio‐Villafán *et al.*, 2018). Adults were fed a 1 : 3 mixture of hydrolyzed protein and sugar, with water provided *ad libitum*.

### Genetic and fitness analysis of mutations

Reciprocal single crosses between the WT strain and the possible mutant strains (♀ WT × ♂ *bp*, ♂ WT × ♀ *bp*, ♀ WT × ♂ *sp* and ♂ WT ×♀ *sp*) were performed, to determined inheritance patterns and potential sex linkage of the mutations. The F_1_ offspring were interbred in groups of five pairs, the F_2_ offspring were meticulously classified using a Zeiss Stemi 2000 stereomicroscope to distinguish phenotypic variations. Potential genetic linkage between the mutations was determined by an initial cross between pure *black pupae* and *sphere pupae* individuals (*bp sp^+^/bp sp^+^* × *bp^+^sp/bp^+^sp*) in a single mating pair. The F_1_ offspring were interbred *en masse*, and the F_2_ offspring were classified by phenotype. To validate the type of linkage between the mutations, one F_1_ male of the GSS generated in this study was evaluated with five *sp* mutant females.

The fitness of the WT strain, homozygous *bp* and *sp* mutants, and the GSS were evaluated using five survival parameters. Egg hatching was quantified as the number of larvae emerging per 100 eggs. Five hundred eggs were incubated at 26 °C for 5 d in a KBF720 Binder bioclimatic chamber. Larval survival (LS) was determined by counting the number of larvae that reached the third instar stage. Pupal survival (PS) was measured as the percentage of larvae that pupated, calculated by dividing the total number of pupae by the total number of larvae. Adult emergence (AE) was determined as the percentage of pupae that successfully developed into adults, dividing the total number of emerged adults by the total pupae. Finally, overall fitness (OF) was calculated using the following formula:

$$OF=\left(\frac{LS}{100}\right)\times \left(\frac{PS}{100}\right)\times \left(\frac{AE}{100}\right).$$

### Development of GSSs based on pupal color

**Fig. 1:**
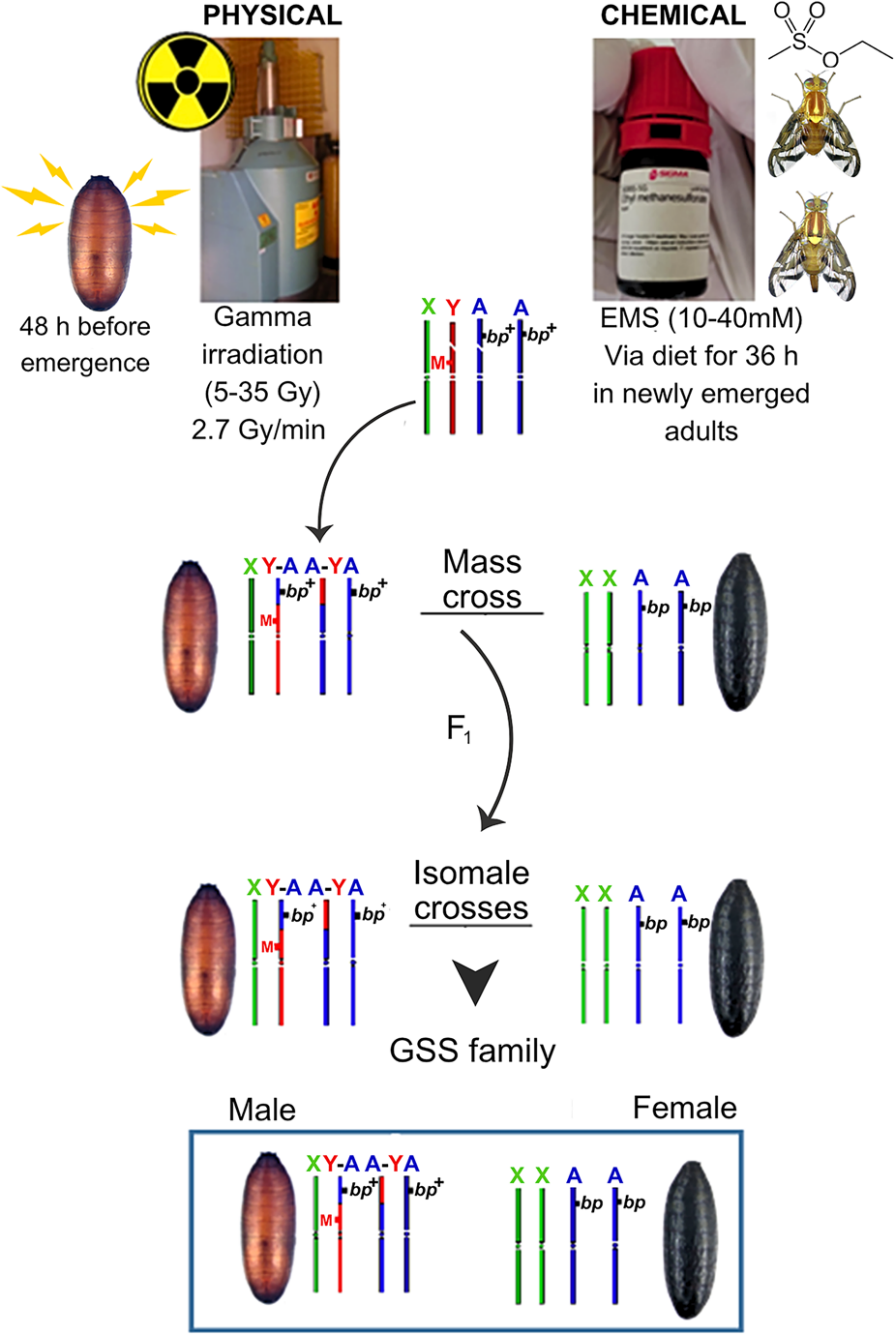
Schematic representation of crosses for the development of a Genetic Sexing Strain for *Anastrepha obliqua* using two induction methods: gamma irradiation and EMS treatment. The approach is based on the translocation of an autosome to the Y chromosome, utilizing the *black pupae* mutation (*bp*) as a morphological marker.

For the development of GSSs, *bp* was selected as a morphological marker due to its easy identification and its higher biological fitness compared to *sp*. Y‐autosome translocations were induced by gamma irradiation and ethyl methanesulfonate (EMS) treatment. In the first approach, WT pupae, 48 h before adult emergence, were exposed to doses of 5, 10, 20, 30, and 35 Gy at a dose rate of 2.7 Gy/min in a Gammabeam™‐127 (Nordion) irradiator with a cobalt^60^ source. In the second approach, newly emerged adults were exposed to 10, 20, 30, and 40 mmol/L EMS via the diet for 36 h. Following treatment, WT males were crossed with homozygous *bp* females (*bp/bp*) at a ratio of 2000 WT males × 4000 *bp/bp* females per experiment. In the F_1_ generation, males were selected to establish 600 independent families, each consisting of one F_1_ male and 15 *bp/bp* females. In the F_2_ generation, families in which males emerged from brown pupae (WT) and females emerged from *bp* were identified as potential carriers of the translocation T(Y;*bp* ^+^)/*bp* (Fig. 1). The selected families were used to develop GSS by crossing brown pupae males with black‐pupae females. In each generation, recombinants were removed (brown pupae females and black pupae males).

### Biological attributes and genetic integrity of GSSs

The biological attributes of the GSS were evaluated through mass crosses under controlled conditions. Each cross included 500 males and 500 females per strain, housed in separate 30 cm^3^ cages. Fertility assessment involved collecting 500 eggs per strain, incubating them at 27 ± 1 °C in a KBF720 Binder bioclimatic chamber for 7 d, and recording the number of hatched eggs. Additionally, 500 neonate larvae per strain were transferred to the artificial diet after 4 d of incubation in aerated water. Ten days later, third‐instar larvae were counted, transferred to sawdust trays for pupation, and monitored for mature pupae and adult emergence.

To assess the feasibility of maintaining the genetic integrity of both GSS, they were reared for more than ten generations using the filter rearing system (FRS) (Fisher & Cáceres, 2000). This involved removing males that emerged from black pupae and females that emerged from brown pupae. Thus, each generation underwent genetic filtering to eliminate recombinants, and maximum egg hatch was evaluated in triplicate, with 100 eggs per replicate per generation. Recombination rate and sex ratio were recorded in each generation.

### Cytogenetic analysis

Mitotic chromosomes were obtained from the third‐instar larval brains of the F_8_ generation of GSS‐selected flies, following the protocol described by García‐Martínez *et al.* (2009). Brain tissue was dissected in a 1% sodium citrate hypotonic solution and incubated for 15 min. Afterward, the tissue was fixed in a freshly prepared 3 : 1 methanol‐acetic acid solution for 5 min. Cells were macerated on clean slides using 60% acetic acid on a 45 °C hot plate until thoroughly dried. Chromosomes were stained for 2 h in a 10% Giemsa solution prepared in sodium phosphate buffer. Microscopic analysis was conducted at 100× magnification using a Carl Zeiss Axioskop 40 microscope, and images were acquired with an AxioCam HRm camera. Only well‐spread metaphases with clearly paired homologous metaphase chromosomes were analyzed. Chromosome length was measured using ImageJ software and expressed as a percentage of the total diploid complement length.

### Data analysis

F_2_ phenotypic frequencies were analyzed using contingency tables and Pearson's chi‐square tests to determine the inheritance pattern of *bp* and *sp* mutations.

The fitness of WT and homozygous mutants (*bp* and *sp*) was evaluated using two statistical approaches, depending on normality and variance homogeneity. When data followed a normal distribution and showed homogeneous variances, ANOVA was applied, followed by Tukey's *post hoc* test to identify specific differences. The Kruskal–Wallis test was used for non‐normal data, followed by Dunn's *post hoc* test with Bonferroni correction (*P* < 0.05). This approach provided a robust and comprehensive statistical framework for evaluating the variables.

The Shapiro–Wilk test was used to assess normality for each variable in both GSS's, and Levene's test was applied to verify the homogeneity of variances. When both assumptions were met (*P* > 0.05), a Student's *t*‐test assuming equal variances was used to compare means between strains. For overall fitness, where the normality assumption was not satisfied for T(Y;*bp* ^+^)/*bp*‐22 (see below), the non‐parametric Mann–Whitney U test was applied. Statistical significance was considered at *P* ≤ 0.05.

All statistical analyses were conducted in R (v4.4.0) using the tidyverse, car, and rstatix packages.

## Results

### Morphological description and genetic analysis of the bp and sp mutants

**Fig. 2:**
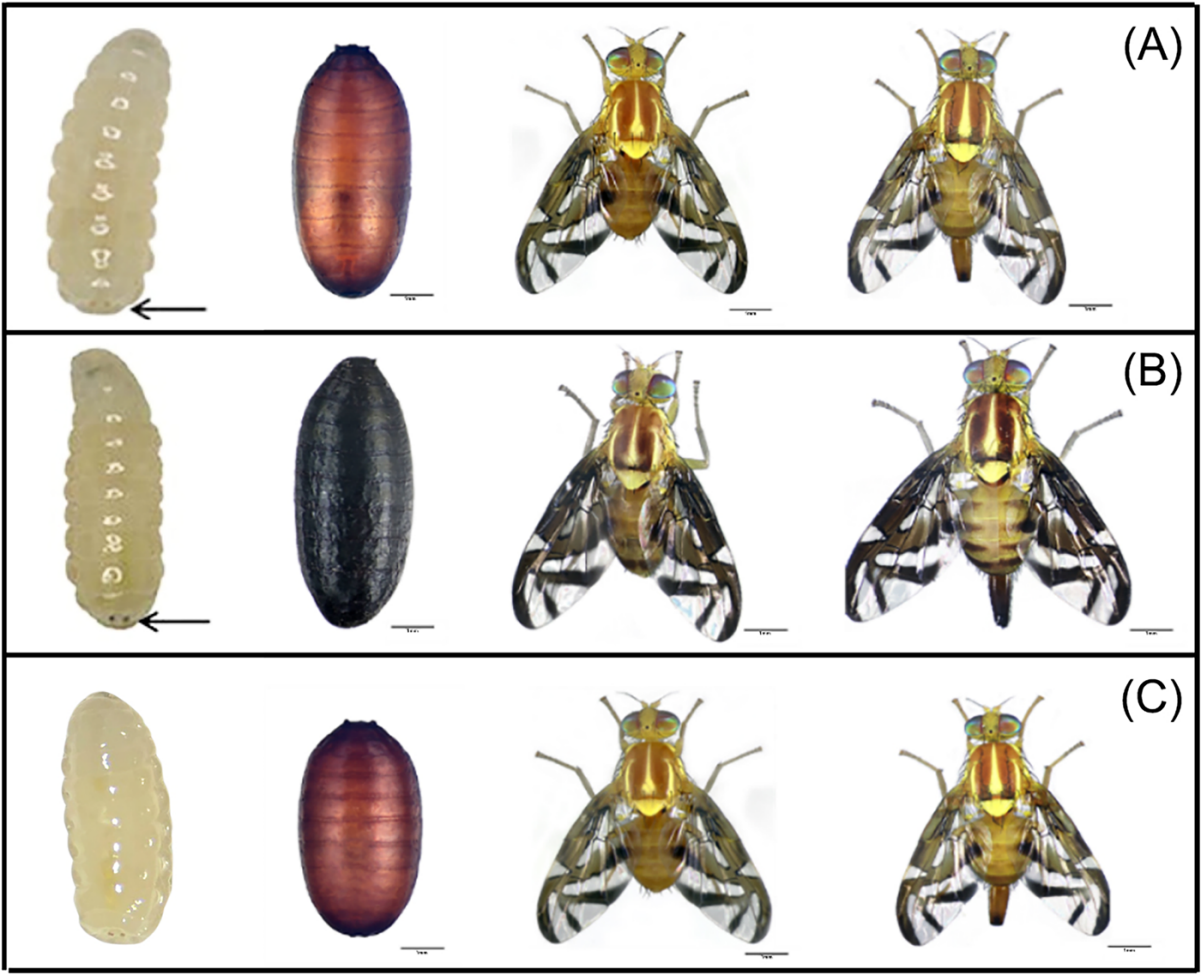
Phenotype of pupae and adult males and females of the wild‐type strain (A) compared to the mutant strains *black pupae* (B) and *sphere pupae* (C).

We identified and characterized two novel mutations: *black pupae* (*bp*) and *sphere pupae* (*sp*), displaying distinct morphological differences. The *bp* mutation has black puparian color, in contrast to the typical wild type reddish‐brown coloration of *A. obliqua* (Fig. 2A). Additionally, *bp* adults exhibit darker thorax, abdomen, and wings than those of the wild‐type strain (Fig. 2B). This phenotype is also evident in the larval stage, as the anal lobes display dark pigmentation. In contrast, the *sp* mutation results in a spherical pupal shape. Adults have a shorter thorax and abdomen than WT individuals, and both share the same normal coloration (Fig. 2C).

### Genetic and fitness analysis of mutations

F_1_ offspring of the four reciprocal crosses between WT and mutant flies displayed a WT phenotype. Inbreeding of the F_1_ progeny in each case resulted in an F_2_ offspring that exhibited both WT and mutant phenotypes. The F_2_ phenotypic frequencies confirmed that phenotype segregation followed the expected pattern for a recessive Mendelian inheritance, showing no significant deviations from a theoretical 3 : 1 WT to mutant ratio (Table 1). This indicated that the inheritance of each mutation is controlled individually by a single autosomal recessive gene. On the other hand, the original cross between the *bp* and *sp* mutant produced only WT offspring in F_1_. This F_1_ offspring was interbred in mass and the F_2_ offspring showed a ratio close to 9 : 3 : 3 : 1 WT to *bp* to *sp* to *bp sp*, indicating that segregation was independent, and that the two loci are on separate chromosomes (Table 1). Backcrossing between a male of T(Y;*bp* ^+^)/*bp*‐22 (see below) and *sp* females, did not show sexing for the *sp* marker, confirming that these two loci (*bp* and *sp*) are not linked (Table 1).

**Table 1:** Inheritance pattern and genetic linkage analysis of *black pupae* and *sphere pupae* mutations

Inheritance crosses (♂ × ♀)		Phenotype of F_2_ offspring				
	No. of cages	WT	*bp*	*sp*	*bp sp*	*χ* ^2^
*bp* × WT	4	4837	1525			3.60^†^
WT × *bp*	4	5953	1919			1.63^†^
*sp* × WT	6	946		282		2.71^†^
WT × *sp*	6	2728		853		2.66^†^
Linkage cross						
*sp* × *bp*	1	225	75	78	23	0.27^‡^
Backcross						
GSS‐22 F_1_ ×*sp*	1	♂ 35 ♀ 46	♂ 26 ♀ 18			

^†^ Hypothesis 3 : 1; *χ* ^2^_0.05, df = 1_ < 3.841.

^‡^ Hypothesis 9 : 3 : 3 : 1; *χ* ^2^_0.05, df = 3_ < 7.82.

The evaluation of biological attributes revealed fitness differences between the WT strain and the *bp* and *sp* mutants, showing significant variation in larval survival (*F* = 8.063, *P* = 0.0018), pupal survival (*χ* ^2^ = 7.51, *P* = 0.0233), adult emergence (*χ* ^2^ = 18.60, *P* < 0.001), and overall fitness (*F* = 25.19, *P* < 0.001). However, fertility among the three strains showed no significant differences (*F* = 2.158, *P* = 0.128). The egg‐to‐larval survival of the *bp* was significantly lower than WT (*P* = 0.0013), while *bp* and *sp* showed no significant differences. Larva to pupal survival differed significantly between *bp* and *sp* (*P* = 0.0348) but remained similar between WT and the two mutants. For pupal to adult survival (adult emergence), *sp* exhibited significantly lower values than *bp* (*P* = 0.0005) and WT (*P* = 0.0007). Finally, overall fitness was significantly lower in *bp* (*P* = 0.0003) and *sp* (*P* < 0.0001) compared to WT, with *sp* displaying the lowest overall fitness relative to *bp* (*P* = 0.0414) (Table 2).

**Table 2:** Percentage values for fitness and survival measures of the *black pupae* (*bp*) and *sphere pupae* (*sp*) mutations compared to the wild‐type strain (WT)

Strain	Fertility	Egg to larvae survival	Larva to pupae survival	Pupa to adults survival	Overall fitness
WT	87.93 ± 0.530 a	66.90 ± 4.715 a	97.12 ± 0.862 ab	86.42 ± 1.488 a	56.3 ± 4.315 a
*bp*	87.33 ± 3.028 a	36.10 ± 4.983 b	97.58 ± 0.928 a	86.24 ± 2.238 a	30.3 ± 4.320 b
*sp*	89.40 ± 3.202 a	47.90 ± 6.541 ab	93.18 ± 1.233 b	36.31 ± 7.361 b	15.3 ± 3.735 c

Values represent mean ± standard error, and different letters within a column indicate statistically significant differences between strains (*P* ≤ 0.05).

### Development of GSSs using the bp marker

Following a crossing and backcrossing scheme (Fig. 1), a total of 17 400 F_1_ males were analyzed using the gamma irradiation induction method, and 12 000 F_1_ males using the EMS induction method. In both induction methods, the highest doses generated Y‐linked translocated lines. Using a 35 Gy irradiation dose, we identified a strain designated as T(Y;*bp* ^+^)/*bp*‐22 (GSS‐22). In contrast, a 40 mmol/L EMS dose produced the strain T(Y;*bp* ^+^)/*bp*‐354 (GSS‐354). No Y‐autosome translocations were recovered at lower irradiation doses (5, 10, 20, or 30 Gy) or at lower EMS concentrations (10, 20, or 30 mmol/L). Both strains exhibited the same phenotypic pattern: males emerged from brown pupae (WT), whereas females emerged from black pupae. However, a proportion of recombinant males was present. Both strains remain under continuous rearing using a generational FRS, with systematic evaluations of their phenotype and fertility parameters.

### Biological attributes and genetic integrity of GSSs

No statistically significant differences were found between the GSS‐22 and GSS‐364 strains for any of the biological attributes evaluated. Student's *t*‐test results were as follows: egg hatching (*t* = 1.92, *P* = 0.090), larval survival (*t* = 1.26, *P* = 0.243), pupal survival (*t* = −1.41, *P* = 0.195), and adult emergence (*t* = 0.67, *P* = 0.521). Also, there were no significant differences in overall fitness between strains (*W* = 17, *P* = 0.345). Nevertheless, a consistent pattern was observed across the biological parameters. Fertility and larval survival showed the lowest values in both strains, with mean egg hatching percentages ranging from 17.8% to 19.8%, and larval survival between 23.8% and 26.0%. In contrast, both egg‐to‐pupa and pupa‐to‐adult survival rates exceeded 90%, indicating high performance in the later developmental stages. The overall fitness remained low in both strains, with average values around 4%, and no significant differences were detected (Table 3).

**Table 3:** Percentage values for survival measures and fitness across the genetic sexing strains, GSS‐22 and GSS‐364

Strain	Fertility	Larvae survival	Egg to pupa survival	Pupa to adult survival	Overall fitness
GSS‐22	19.20 ± 1.30 a	26.00 ± 3.08 a	91.37 ± 3.33 a	93.68 ± 5.18 a	4.4 ± 0.40 a
GSS‐364	17.80 ± 2.17 a	23.80 ± 2.39 a	94.02 ± 2.54 a	91.31 ± 5.96 a	3.4 ± 0.37 a

Values represent mean ± standard error, and different letters within a column indicate statistically significant differences between strains (*P* ≤ 0.05).

To ensure genetic integrity, an FRS was applied to the GSS‐22 and GSS‐364 strains for 14 and 11 generations, respectively. The GSS‐22 parental cross began with 25 males and 35 females, and the number progressively increased to 1850 males and 1740 females by F_14_ (Table 4). The GSS‐364 parental cross began with 15 males and 18 females, by F_11_, it had grown to 1449 males and 1392 females (Table 5). Population growth in both GSS correlated with the low hatch rates observed in early generations. Fertility increased in both GSS lines across generations, reaching average maximum egg hatch rates of 23.33% for GSS‐22 and 20.67% for GSS‐364. Compared to the F_1_, both GSS showed significant increases in fertility. GSS‐22 rose from 5% in F_1_ to 23.33% in F_14_ (Table 4), while GSS‐364 exhibited a more moderate increase, from 14.33% in F_1_ to 22% in F_11_ (Table 5).

**Table 4:** Phenotypes, fertility and recombination obtained from filter rearing system applied to GSS‐22 obtained through radiation (35 Grays)

Generation	Brown pupae	Brown pupae adults		Black pupae	Black pupae adults		Fertility (mean ± SD) (%)	Recombinants Brown pupae (%)	Recombinants Black pupae (%)
		♂	♀		♂	♀			
Parental	25	25	0	38	7	31	4.33 ± 1.53	0.00	11.11
F_1_	24	23	0	49	5	43	5.00 ± 1.00	0.00	7.04
F_2_	125	117	0	159	44	94	9.33 ± 1.15	0.00	17.25
F_3_	259	204	0	281	58	175	8.33 ± 1.53	0.00	13.27
F_4_	258	245	0	285	78	204	10.00 ± 2.00	0.00	14.80
F_5_	247	240	0	299	40	245	11.67 ± 2.08	0.00	7.62
F_6_	217	196	0	275	54	207	13.33 ± 2.31	0.00	11.82
F_7_	350	347	0	393	77	299	16.00 ± 1.73	0.00	10.65
F_8_	390	375	0	428	53	375	15.33 ± 1.15	0.00	6.60
F_9_	503	478	0	625	82	543	20.67 ± 3.06	0.00	7.43
F_10_	1002	973	0	1189	221	875	20.67 ± 3.61	0.00	10.68
F_11_	829	817	0	957	102	839	21.33 ± 2.52	0.00	5.80
F_12_	1253	1240	0	1429	295	1125	21.67 ± 3.06	0.00	11.09
F_13_	1425	1403	2	1687	235	1411	23.00 ± 2.00	0.07	7.71
F_14_	1865	1850	3	2068	328	1740	23.33 ± 0.58	0.08	8.37

**Table 5:** Phenotypes, fertility, and recombination obtained from the filter rearing system applied to GSS‐364 obtained through EMS (40 mmol/L)

Generation	Brown pupae	Brown pupae adults		Black pupae	Black pupae adults		Fertility (mean ± SD) (%)	Recombinants Brown pupae (%)	Recombinants Black pupae (%)
		♂	♀		♂	♀			
Parental	18	15	0	23	3	18	13 ± 2.65	0.00	8.33
F_1_	32	31	0	62	9	41	14.33 ± 3.21	0.00	11.11
F_2_	35	31	0	42	5	35	10.30 ± 1.53	0.00	7.04
F_3_	85	77	1	92	6	84	22.33 ± 3.51	0.60	3.59
F_4_	112	105	0	128	12	110	20.67 ± 4.51	0.00	5.29
F_5_	120	115	0	153	14	139	16.00 ± 2.00	0.00	5.22
F_6_	540	523	4	745	89	629	17.00 ± 1.73	0.32	7.17
F_7_	825	798	10	983	245	629	21.67 ± 3.06	0.59	14.65
F_8_	832	790	35	968	163	697	19.33 ± 1.53	5.82	9.88
F_9_	1025	760	148	1325	320	925	21.67 ± 1.15	6.87	15.96
F_10_	1324	1197	123	1425	247	1489	22.33 ± 3.06	4.02	8.42
F_11_	1512	1449	53	1759	367	1392	22.00 ± 2.00	1.63	11.44

In GSS‐22, the male recombination frequency fluctuated between 5.80% and 17.25% across generations, while recombinant females did not appear until F_13_, when the population was scaled to 1000 pairs per cage, reaching 0.065% and slightly increasing to 0.077% in F_14_ (Table 4). In contrast, GSS‐364 displayed both male and female recombinants from early generations. The male recombination frequency ranged from 3.59% to 15.96%, while female recombinants reached up to 6.87% in F_9_ (Table 5). These results suggest that the integrity of genetic can be maintained by the application of an FRS in the GSS‐22 better than GSS‐364, over the generations evaluated.

### Cytogenetic analysis

**Fig. 3:**
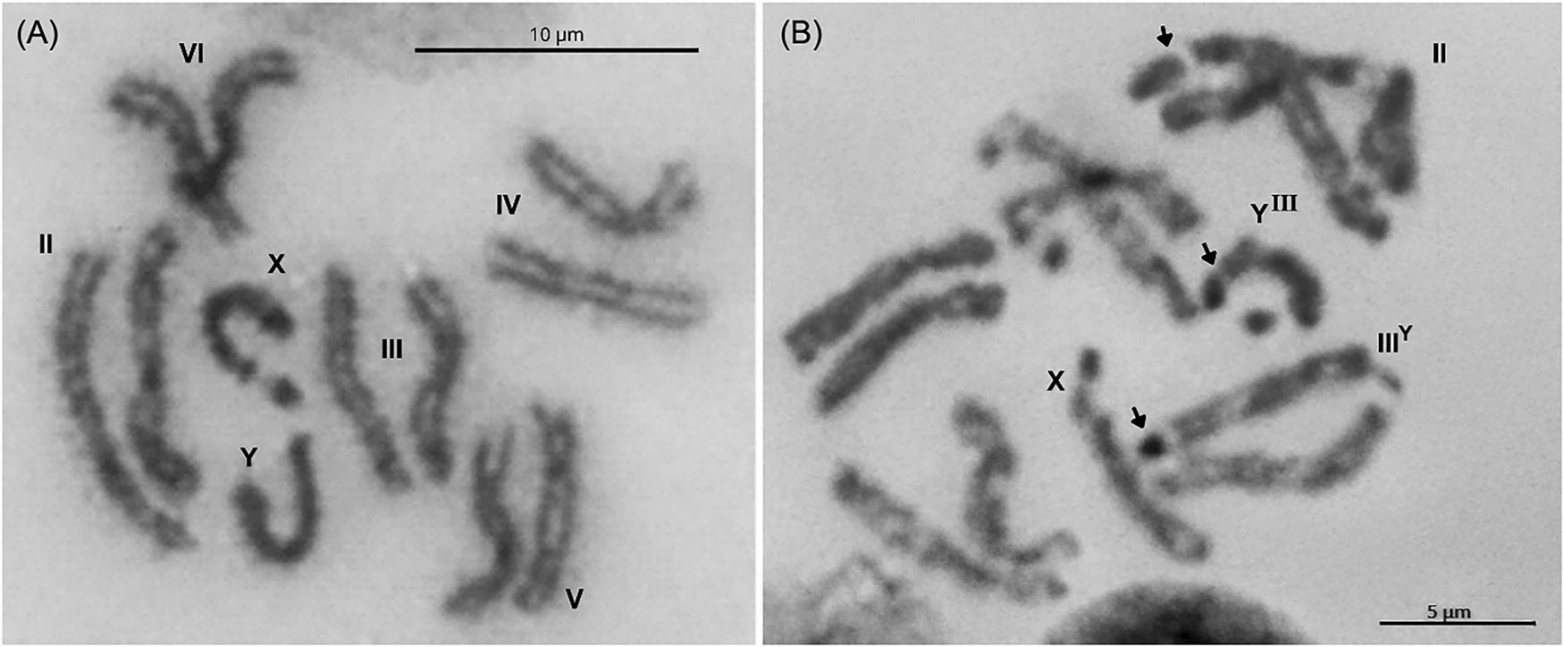
Cytogenetic representation of (A) mitotic metaphase chromosomes from the brain ganglia of a third‐instar male larva of the wild‐type *A. obliqua* strain and (B) mitotic metaphase chromosomes of the GSS‐22. The three arrowheads highlight chromosomal regions that differ from the wild‐type complement.

Since GSS‐22 showed the greatest stability in integrity across generations using FRS, it was selected for cytogenetic analysis, and 300 metaphase spreads of mitotic chromosomes were prepared. We selected 39 with well‐extended chromosomes paired with their homologs to ensure the highest measurement accuracy. Among these, 28 (71.7%) exhibited differences between homologs of chromosome pair 3 (Fig. 3). The X chromosome and chromosome 2, recognizable by their distinctive length and morphology, served as references to validate the analysis results. However, cytogenetic analysis also revealed chromosomal breakage in other regions (Fig. 3) and less frequent alterations in other autosomes. In total, we identified modifications in chromosome pair 4 (7 metaphases; 17.9%), chromosome 5 (4 metaphases; 10.2%), and chromosome 6 (1 metaphase; 2.5%) (Table 6).

**Table 6:** Comparison of chromosome length and relative differences between the wild‐type strain and the translocated strain GSS‐22

CR	Wild type				T(Y/*bp* ^+^)‐22			
	TL (µm)	DL (µm)	LR (%)	DRL (%)	TL (µm)	DL (µm)	LR (%)	DRL (%)
2	12.64 ± 2.27	0.27 ± 0.18	11.32 ± 0.73	0.24 ± 0.14	11.68 ± 2.17	0.13 ± 0.58	11.20 ± 0.72	0.12 ± 0.09
	12.37 ± 2.66		11.08 ± 0.74		11.55 ± 2.16		11.08 ± 0.71	
3	9.70 ± 2.12	0.21 ± 0.16	8.67 ± 0.46	0.19 ± 0.13	9.35 ± 1.62	1.14 ± 0.89	8.98 ± 0.42	1.07 ± 0.78
	9.49 ± 2.02		8.48 ± 0.49		8.22 ± 1.49^†^		7.91 ± 0.81	
4	9.23 ± 2.01	0.17 ± 0.13	8.26 ± 0.29	0.15 ± 0.09	8.74 ± 1.59	0.26 ± 0.35	8.38 ± 0.35	0.25 ± 0.30
	9.07 ± 1.95		8.11 ± 0.30		8.48 ± 1.59		8.13 ± 0.51	
5	8.80 ± 1.98	0.18 ± 0.11	7.86 ± 0.38	0.17 ± 0.11	8.48 ± 1.62	0.34 ± 0.57	7.91 ± 0.35	0.32 ± 0.49
	8.62 ± 1.97		7.70 ± 0.39		7.91 ± 1.45		7.59 ± 0.49	
6	8.14 ± 1.66	0.14 ± 0.11	7.31 ± 0.39	0.13 ± 0.11	7.71 ± 1.46	0.21 ± 0.29	7.39 ± 0.44	0.20 ± 0.27
	8.00 ± 1.69		7.18 ± 0.40		7.51 ± 1.45		7.19 ± 0.50	
X	8.63 ± 1.75	‒	7.75 ± 0.75	‒	8.02 ± 1.18	‒	7.77 ± 0.78	‒
Y	6.99 ± 1.44	‒	6.29 ± 0.61		6.65 ± 0.92^†^		6.45 ± 0.59	

CR, chromosome; TL, total length; DL, difference in length; LR, relative length; DRL, difference in relative length.

^†^Possibly involved in translocation.

## Discussion

In this study, two pupal mutations of *A. obliqua* that affect the color (*black pupae*, *bp*) and shape (*sphere pupa*, *sp*) of the puparium were isolated and characterized. GSSs were constructed for the first time for this pest species. The *bp* mutation showed a phenotype and inheritance similar to the *black pupae* mutation of *A. ludens* and *A. fraterculus* (Zepeda‐Cisneros *et al.*, 2014; Meza *et al.*, 2020), which is an efficient visual marker for sex differentiation compared to *sp*, whose phenotypic detection is more challenging under mass‐rearing conditions (Rössler, 1979). Both mutations, *bp* and *sp*, follow a recessive Mendelian inheritance pattern, aligning with the expected phenotypic ratios (Ward *et al.*, 2021; Paulo *et al.*, 2025). Linkage analysis indicated that the genes responsible for these mutations are on different chromosomes.

Biological performance analyses revealed that both mutations significantly affect individual fitness. The *bp* mutation reduces larval survival compared to the WT strain, while *sp* shows the lowest fitness among all evaluated lines. These results align with the pleiotropic effects associated with these mutations, which, in addition to altering the phenotype, may interfere with various physiological and behavioral processes (Takahashi, 2013; Meza *et al.*, 2019).

We selected the *bp* mutation for the development of a GSS, which enabled the construction of the GSS‐22 and GSS‐354 through irradiation (35 Gy) and EMS treatment (40 mmol/L), respectively. Both methodologies generated functional lines, although they initially showed reductions in biological parameters and fitness compared to the WT and *bp* lines. These reductions are characteristic of translocated lines and are consistent with previous studies in other tephritid species (Cáceres *et al.*, 2004; Vreysen *et al.*, 2021).

Although precise thresholds for optimal irradiation remain undefined, studies on *A. obliqua* have shown that doses ≥ 25 Gy induce high sterility, while doses ≥ 60 Gy markedly reduce viability with 98% sterility and disrupt behavior (Toledo *et al.*, 2004; Gallardo‐Ortiz *et al.*, 2018). Consistent with these findings, exposure of pupae to 35 Gy induced the targeted Y‐autosome translocation but yielded very low F_1_ egg‐hatch rates, 4.0% ± 0.5% in GSS‐22 versus 13.2% ± 1.1% in GSS‐364 (EMS) (Meza *et al.*, 2019). Translocation‐based GSS are inherently semi‐sterile, under Mendelian segregation, only half of the gametes inherit the balanced Y‐autosome complement required for viability (Robinson, 1976), and pleiotropic or off‐target effects often reduce hatch rates. For example, male‐linked translocation in *Culex pipiens* maintains egg‐hatch rates of only ∼15% (Laven & Aslamkhan, 1970; Robinson, 1976).

Despite this initial suppression, both lines demonstrated progressive increases in egg‐hatch rates, rising from 5.0% (F_1_) to 23.3% (F_14_) in GSS‐22, and from 14.3% (F_1_) to 22.0% (F_11_) in GSS‐364. To our knowledge, no prior Tephritidae GSS study has reported such detailed, generation‐by‐generation hatch data documenting fertility recovery. These findings support the hypothesis that homogeneous rearing combined with a filter rearing system gradually eliminates unbalanced chromosomal rearrangements and mitigates the fitness costs inherent to Y‐autosome translocations (Rössler, 1979; Toledo *et al.*, 2004; Zepeda‐Cisneros *et al.*, 2014; Franz, 2001).

Beyond fertility, the two lines diverged in genetic stability. The irradiation‐induced GSS‐22 exhibited greater long‐term genetic stability, characterized by a progressive decline in male recombinant frequency and the emergence of female recombinants in generations F_13_ (0.065%) and F_14_ (0.077%). This result may be attributed to the effects of double‐strand breaks induced by irradiation, which facilitate the formation of targeted chromosomal rearrangements (Robinson, 1976; Robinson, 2002b).

In contrast, the EMS‐derived GSS‐364 line displayed lower genetic stability, marked by a higher frequency of male recombinants in early generations and a notable increase in female recombinants starting in F_7_. This phenomenon may stem from EMS's mode of action, which induces point mutations by alkylating nitrogenous bases and causing genomic alterations. Thus, unlike irradiation, EMS leads to the accumulation of recessive lethal and sub‐lethal alleles that are unmasked under successive inbreeding and at higher doses, can also provoke small insertions/deletions and local chromosomal lesions, further undermining the production of balanced gametes (Ohnishi, 1977; Sega, 1984; Kodym & Afza, 2003; Chen *et al.*, 2023).

The black pupae phenotype results from mutations in *ebony*, a key gene in melanin synthesis that regulates the conversion of dopamine into *N‐β‐alanyl dopamine* (NBAD), the precursor of yellow sclerotin in the cuticle. Mutations in *ebony* disrupt this pathway, redirecting dopamine toward melanin production and resulting in individuals with hyperpigmented cuticles (Paulo *et al.*, 2025). The high conservation of *ebony* across different *Anastrepha* species suggests that there is strong evolutionary pressure to maintain its function. In *A. ludens*, this gene has been mapped to mitotic chromosome 2 (Paulo *et al.*, 2025), whereas in *A. obliqua*, the genomic assembly *GCF_027943255.1* places it on chromosome 1 (*XM_054870789.1*). This chromosome appears homologous to chromosome 2 in *A. ludens*, exhibiting a similar chromosomal localization in both species.

Despite this evidence, cytogenetic analysis of the GSS‐22 revealed that the predominant translocation involves chromosome 3, given the high frequency of alterations in its length. However, detecting breaks in other chromosomes suggests a more complex chromosomal arrangement. Although the analyzed metaphases did not show frequent morphological differences in other autosomes, the possibility of undetected rearrangements through mitotic chromosome analysis highlights the limitations of this approach for characterizing chromosomal translocations. This limitation is particularly relevant in species like *A. obliqua*, whose autosomes exhibit similar morphology (Ibáñez‐Palacios *et al.*, 2010; Zepeda‐Cisneros *et al.*, 2014).

Previous studies on tephritids have shown that accurately characterizing translocations requires complementary polytene chromosome analysis (Zacharopoulou *et al.*, 2017). Integrating this approach with genomic analyses would improve the resolution of chromosomal rearrangements in GSS‐22 and help clarify discrepancies observed in mitotic analyses. Additionally, sequential back‐crossing of *bp*–Y translocated males with *bp* females, coupled with stringent selection for high male fertility, should mitigate irradiation‐induced genomic instability by accelerating the loss of secondary chromosomal rearrangements unlinked to the breakpoint while preserving the translocation (Hospital, 2005; Isasawin *et al.*, 2014; Ntoyi *et al.*, 2022).

This study provides the first report of the *bp* and *sp* mutations in *A. obliqua* and their application in GSS development. GSS‐22, generated through irradiation, exhibited greater genetic stability and functionality than GSS‐364, which resulted from EMS treatment. Discrepancies between cytogenetic and genomic data underscore the need for integrated strategies to resolve the chromosomal structure of GSS‐22. Our immediate aims are to enhance fertility through directed selection and to conduct semi‐mass‐rearing trials that evaluate key quality‐control metrics alongside genetic stability, utilizing optimized filter‐rearing protocols to minimize female recombinants. This study represents a significant advance in *A. obliqua* genetic control and the potential for their implementation in area‐wide sterile‐insect technique programs.

## Disclosure

All the authors confirm there is no conflict of interest.
